# The Carboxyl Terminal Regions of P0 Protein Are Required for Systemic Infections of Poleroviruses

**DOI:** 10.3390/ijms23041945

**Published:** 2022-02-09

**Authors:** Xin Zhang, Mamun-Or Rashid, Tian-Yu Zhao, Yuan-Yuan Li, Meng-Jun He, Ying Wang, Da-Wei Li, Jia-Lin Yu, Cheng-Gui Han

**Affiliations:** 1 State Key Laboratory for Agro-Biotechnology and Ministry of Agriculture Key Laboratory of Pest Monitoring and Green Management, College of Plant Protection, China Agricultural University, Beijing 100193, China; zhangxinhn@cau.edu.cn (X.Z.); mamun_1961@yahoo.com (M.-O.R.); hunan_lyy@cau.edu.cn (Y.-Y.L.); 18864805798@163.com (M.-J.H.); yingwang@cau.edu.cn (Y.W.); Lidw@cau.edu.cn (D.-W.L.); yujl@cau.edu.cn (J.-L.Y.); 2 China National Center for Biotechnology Development, Beijing 100039, China; ty_zhao@163.com

**Keywords:** Brassica yellows virus, *Potato leafroll virus*, P0 protein, truncated mutation, viral suppressor of RNA silencing, virus systemic infection

## Abstract

P0 proteins encoded by poleroviruses Brassica yellows virus (BrYV) and *Potato leafroll virus* (PLRV) are viral suppressors of RNA silencing (VSR) involved in abolishing host RNA silencing to assist viral infection. However, other roles that P0 proteins play in virus infection remain unclear. Here, we found that C-terminal truncation of P0 resulted in compromised systemic infection of BrYV and PLRV. C-terminal truncation affected systemic but not local VSR activities of P0 proteins, but neither transient nor ectopic stably expressed VSR proteins could rescue the systemic infection of BrYV and PLRV mutants. Moreover, BrYV mutant failed to establish systemic infection in DCL2/4 RNAi or RDR6 RNAi plants, indicating that systemic infection might be independent of the VSR activity of P0. Partially rescued infection of BrYV mutant by the co-infected PLRV implied the functional conservation of P0 proteins within genus. However, although C-terminal truncation mutant of BrYV P0 showed weaker interaction with its movement protein (MP) when compared to wild-type P0, wild-type and mutant PLRV P0 showed similar interaction with its MP. In sum, our findings revealed the role of P0 in virus systemic infection and the requirement of P0 carboxyl terminal region for the infection.

## 1. Introduction

RNA silencing is a natural host antiviral defense pathway at the nucleic acid level [1,2]. It occurs through the processing of double-stranded RNAs into complementary short (21–24 nucleotides) interfering RNAs (siRNAs) by the Dicer-like enzyme RNAse III [3,4]. These siRNAs are loaded into RNA-induced silencing complexes to degrade viral RNAs [5,6]. Resulting fragments are converted into dsRNAs with the help of RNA-dependent RNA polymerases (RDRs) and the cofactor suppressor of gene silencing 3 (SGS3), which is encoded by the plant host, to yield secondary siRNAs [7,8]. RDR1, RDR2 and RDR6 play important roles in siRNA production through viral infections [7,9]. The P0 protein of *Turnip yellows virus* (formerly known as *Beet western yellows virus* FL1 strain) was the first VSR identified in the genus *Polerovirus* [10]. In the past decade, P0 proteins from a number of economically important poleroviruses, including *Beet chlorosis virus*, *Beet mild yellowing virus*, Brassica yellows virus (BrYV), *Cotton leafroll dwarf virus*, *Cucurbit aphid-borne yellows virus*, *Melon aphid-borne yellows virus*, Pea mild chlorosis virus, *Sugarcane yellow leaf virus* (SCYLV), *Wheat yellow dwarf virus*-GPV isolate and *Potato leafroll virus* (PLRV), have been reported to be involved in suppressing RNA silencing [11,12,13,14,15,16,17,18,19]. The amino acid sequence identity level among these *Polerovirus* P0s is very low [19]. In spite of the considerable sequence differences, P0 proteins from different poleroviruses still share several motifs or region, which are essential for suppression of RNA silencing. These include the F-box-like motif, G139/W140/G141-like motif and the C-terminal conserved region. In addition, the N-terminal truncation of some P0 abolish their suppressor activity, while a certain range of C-terminal truncations only affects the systemic VSR activity level [11,19]. Recent studies demonstrated that polerovirus-encoded P0 proteins can target the membrane-bound ARGONAUTE1 (AGO1) and induce an ER-derived autophagy degradation pathway for their degradation [10,20,21,22].

In addition to suppressing host antiviral RNA silencing, many VSRs are multifunctional and play important roles in viral replication, coating, movement and pathogenesis [23]. Some of them are reported to be related to systemic movement of viruses. When P14, a VSR of BNYVV, is mutated, BNYVV is unable to spread systemically [24]. It has been shown for a long time that P0 of poleroviruses is indispensable for the virus accumulation [25,26]. In our previous studies, we also found that mutants of BrYV or PLRV in which the VSR functions of P0 are abolished, have much lower virus accumulations in the inoculated leaves and are undetectable in the upper leaves of plants, demonstrating that P0 is essential for an efficient systemic infection of poleroviruses [12,27]. Our recent work revealed that P0^Br^ stabilizes itself through interaction with NbSKP1 and interacts with NbRAF2 to facilitate virus infection [12,28]. However, the relationship between systemic movement of polerovirus and various functions of P0 is still unknown. The region in P0, which is dispensable for systemic movement, is unclear.

For systemic infection and symptom expression of poleroviruses, the P3a, CP and C-terminal region in RTP are reported to be important [29,30,31,32]. According to other research, the PLRV mutant, which does not translate the P17 (MP), can still spread systemically from inoculated leaves in *Nicotiana* spp., showing that P17 is not necessary for the movement of PLRV in *Nicotiana* spp. However, this PLRV mutant is unable to initiate systemic infection in potato and *P. floridana*, indicating a host-dependent requirement for the movement protein in virus movement [33]. It will be interesting to analyze whether P0 proteins co-function with these movement-related proteins during virus infection.

Here, by screening the VSR activities of a series of C-terminal truncated P0 mutants, we identified the regions in C-termini of BrYV P0 and PLRV P0 that are essential for the systemic, but not the local VSR activity. Based on these results, cDNA clones of BrYV and PLRV mutants, which encode the selected C-terminal truncated P0, were generated. The results of inoculation experiments showed that the short C-terminal 15 amino acid residues of BrYV and PLRV P0s were required for systemic viral infections. Neither ectopic stable nor transient expressions of VSR proteins rescued the systemic infection of BrYV mutant. A co-immunoprecipitation analysis further revealed that both the P0 C-terminal truncated mutants and wild types of BrYV and PLRV interacted with their MPs.

## 2. Results

### 2.1. Screening Suitable Truncated Regions in the C-Termini of P0^Br^ and P0^PL^ That Retained Local but Not Systemic VSR Activity

**Figure 1:**
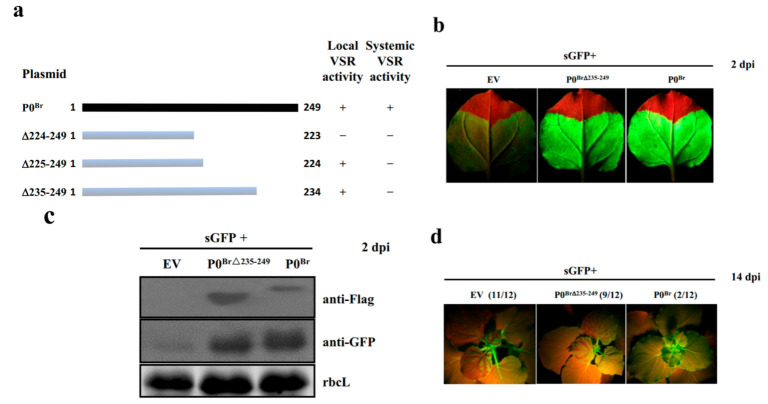
Verification of P0^Br∆235–249^ VSR activity. (**a**) Schematic representation of P0^Br^ and P0^Br^ truncated mutants used to screen for a suitable region that abolished RNA-silencing suppressor activity. +: Retained; −: Abolished. (**b**) Analysis of local RNA-silencing suppression of P0^Br∆235–249^. GFP was expressed in co-infiltrated wild-type *N. benthamiana* leaf patches at 2 dpi under UV illumination. The left sides of leaves are expressing GFP of EV (negative control), the right sides are expressing GFP of wild-type P0^Br^ (positive control). (**c**) Western blotting analyses of GFP and 3Flag-tagged P0^Br^ or P0^Br∆235–259^ in co-infiltrated leaf patches of *N. benthamiana* using a GFP polyclonal antibody (anti-GFP) and a Flag monoclonal antibody (anti-Flag), respectively. Stained rubisco is shown to indicate equal lane loading. (**d**) Analysis of systemic RNA-silencing suppression by P0^Br∆235–249^. GFP was expressed in the upper leaves of co-infiltrated *N. benthamiana* line 16c plants at 14 dpi under UV illumination. The numbers denote silencing plants with the total number of plants tested.

Previously, we revealed that deletion of 25 amino acids in the C-terminus of BrYV P0 abolished its systemic RNA silencing suppression activity without affecting the local RNA silencing suppression [12]. To analyze the function of P0 C-terminus during virus infection, we need a BrYV mutant which encodes the C-terminal truncated P0 without affecting translation product of the overlapped ORF1. For this purpose, we deleted 15 amino acids in the C-terminus of P0^Br^ (P0^Br∆235–249^) (Figure 1a) and evaluated suppressor activity of this mutant by *Agrobacterium*-mediated transient co-expression assay with pGDG (for the expression of GFP) in *N. benthamiana* leaves. At 2 dpi, the leaf patch co-infiltrated with P0^Br∆235–249^ mutant exhibited strong GFP fluorescence similar to that of wild-type P0^Br^ (Figure 1b). Western blotting results further confirmed that GFP protein accumulation was consistent with GFP fluorescence levels (Figure 1c). To analyze the systemic silencing suppression ability of P0^Br∆235–249^, we performed *Agrobacterium*-mediated co-expression assay in GFP transgenic *N. benthamiana* line 16c. At 14 dpi, the plants co-infiltrated with GFP and empty vector (EV) showed systemic silencing of GFP. Systemic leaves of plants co-infiltrated with GFP and wild-type P0^Br^ still exhibited green fluorescence, indicating P0^Br^-mediated suppression of systemic RNA-silencing. However, the plants co-infiltrated with GFP and P0^Br∆235–249^ showed systemic RNA silencing (Figure 1d).

**Figure 2:**
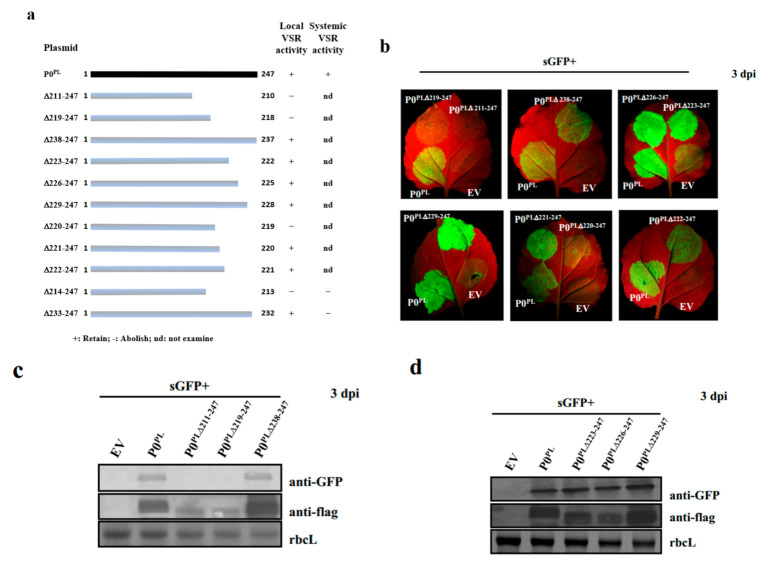

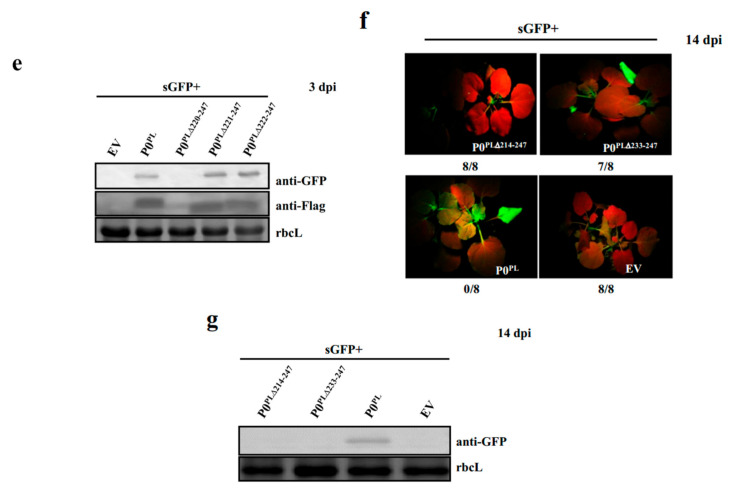
Screening for a suitable truncated region in the C-terminus of P0^PL^ that abolished local or systemic RNA-silencing suppressor activity. (**a**) Schematic representation of P0^PL^ and P0^PL^ truncated mutants used to screen for a suitable region that retained or abolished RNA-silencing suppressor activity. +: Retained; −: Abolished; nd: not examined. (**b**) Analysis of local RNA-silencing suppression. GFP was expressed in co-infiltrated wild-type *N. benthamiana* leaf patches at 3 dpi under UV illumination. The lowermost right sides of leaves expressed GFP of EV (negative control), the lowermost left sides expressed GFP of wild-type P0^PL^ (positive control) and the uppermost right and left sides expressed GFP of the P0^PL^ truncated mutants. (**c**–**e**) Western blotting analyses of GFP and 3Flag-tagged P0^PL^ or P0^PL^ truncated mutants in co-infiltrated leaf patches of *N. benthamiana* using a GFP polyclonal antibody (anti-GFP) and a Flag monoclonal antibody (anti-Flag), respectively. Stained rubisco is shown to indicate equal lane loading. (**f**) Analysis of systemic RNA-silencing suppression. GFP was expressed in the systemically infected leaves of respective co-infiltrated *N. benthamiana* line 16c plants at 14 dpi under UV illumination. Designations are specified at the lowermost right sides of pictures, whereas bottom numbers denote silencing ratios. (**g**) Western blotting analyses of GFP from the P0^PL^ or P0^PL^ truncated mutants in the systemically infected leaves of respective *N. benthamiana* line 16c plants using a GFP polyclonal antibody (anti-GFP). Stained rubisco is shown to indicate equal lane loading.

To screen the suitable regions in the C-terminus of P0^PL^ that abolished local VSR activity, nine P0^PL^ C-terminal truncated mutants (P0^PL∆211–247^, P0^PL∆219–247^, P0^PL∆220–247^, P0^PL∆221–247^, P0^PL∆222–247^, P0^PL∆223–247^, P0^PL∆226–247^, P0^PL∆229–247^, P0^PL∆238–247^) were constructed (Figure 2a) and independently co-infiltrated with pGDG into *N. benthamiana* leaves. Negligible GFP fluorescence could be observed in the leaf patches co-infiltrated with GFP and three mutants (P0^PL∆211–247^, P0^PL∆219–247^, P0^PL∆220–247^) or EV, whereas leaf patches co-infiltrated with the mutant P0^PL∆221–247^ and the rest of the five mutants exhibited strong GFP fluorescence similar to wild-type P0^PL^ at 3 dpi (Figure 2b). Subsequent Western blotting results showed that GFP protein accumulation was consistent with GFP fluorescence levels (Figure 2c–e). These results indicated that a deletion more than 27 amino acids in the C-terminus of P0^PL^ abolished its local VSR activity. To determine the systemic VSR activity of P0^PL^ and its mutants, *N. benthamiana* line 16c plants were co-infiltrated with pGDG and truncated P0 mutants (one containing local VSR activity (P0^PL∆233–247^) and another that abolished local VSR activity (P0^PL∆214–247^)). At 14 dpi, upper leaves of the respective co-infiltrated plants for both truncated P0 mutants, or EV, displayed negligible amounts of GFP fluorescence, whereas the plants co-infiltrated with GFP and wild-type P0^PL^ showed a strong GFP fluorescence (Figure 2f). Western blotting results further confirmed that GFP protein accumulation was consistent with GFP fluorescence levels (Figure 2g), indicating that both of the truncated mutants abolished their systemic VSR activity. In sum, the mutant P0^PL∆214–247^ lost both local and systemic VSR activities, and P0^PL∆233–247^ retained local but lost systemic VSR activities.

### 2.2. C-Terminal 15 Amino Acid Residues Truncations in the P0 Proteins of BrYV and PLRV Had Significant Impacts on Systemic Viral Infections in Plants

**Figure 3:**
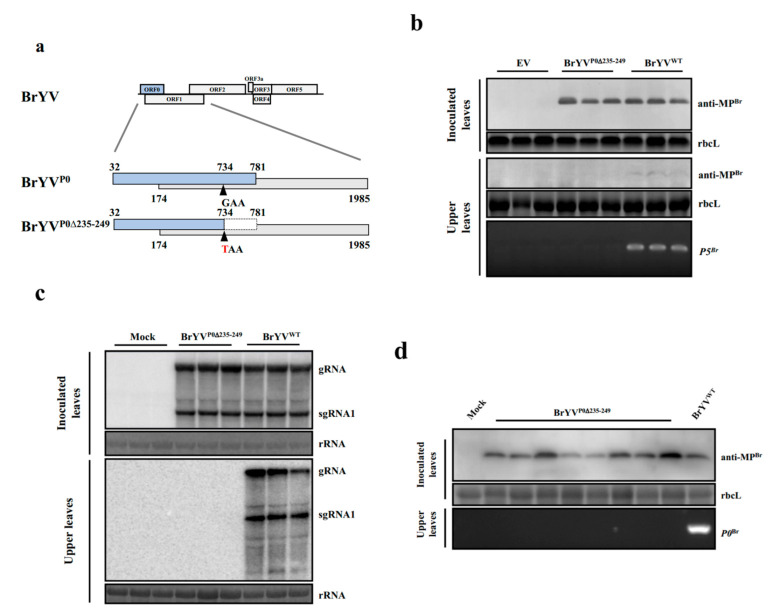
BrYV^P0∆235–249^ mutant virus showing the capability to produce a systemic infection. (**a**) BrYV^P0∆235–249^ mutation is incorporated into the P0 coding sequence without disrupting the P1 sequence. (**b**) Protein determination in virus-inoculated leaves at 2 dpi and the upper leaves of *N. benthamiana* plants at 14 dpi by Western blotting analysis with a BrYV-MP-specific antibody (anti-MP^Br^). Stained rubisco is shown to indicate equal lane loading. RNA determination from the upper leaves of *N. benthamiana* at 14 dpi as assessed by RT-PCR using P5^Br^ gene-specific primers. (**c**) Northern blotting analysis of viral RNAs from inoculated leaves at 2 dpi and upper leaves of *N. benthamiana* plants at 14 dpi. (**d**) Protein determination in virus-inoculated *Arabidopsis thaliana* leaves at 5 dpi by Western blotting analysis with a BrYV-MP-specific antibody (anti-MP^Br^). RNA determination in the upper leaves of *Arabidopsis thaliana* plants at 14 dpi as assessed by RT-PCR using P0^Br^ gene-specific primers.

Based on the above results, a BrYV mutant cDNA clone, named BrYV^P0∆235–249^, was generated by introducing a premature stop codon at position nt-734 in the ORF0. This mutant encodes a systemic-VSR-defective version of P0 without altering the translation products of the overlapped ORF1 (Figure 3a). To address the impact of the mutation on BrYV RNA and protein accumulation, *N. benthamiana* plants were inoculated with BrYV^P0∆235–249^ by Agro-infiltration. At 2 dpi, Western and Northern blotting analyses showed that the BrYV mutant accumulation level in inoculated leaves was similar to that of wild-type (Figure 3b,c). To determine the impact of the mutation on BrYV systemic infections in *N. benthamiana* plants, extracts from the upper leaves of *N. benthamiana* plants were subjected to RT-PCR, Western and Northern blotting at 14 dpi. Only the wild-type virus caused systemic infections, whereas the mutant BrYV^P0∆235–249^ was not able to infect upper leaves of inoculated plants (Figure 3b,c). Similarly, the mutant BrYV^P0∆235–249^ could not systemically infect *Arabidopsis thaliana* plants (Figure 3d).

**Figure 4:**
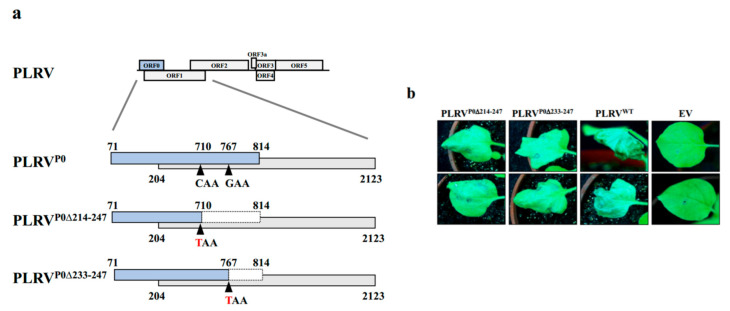

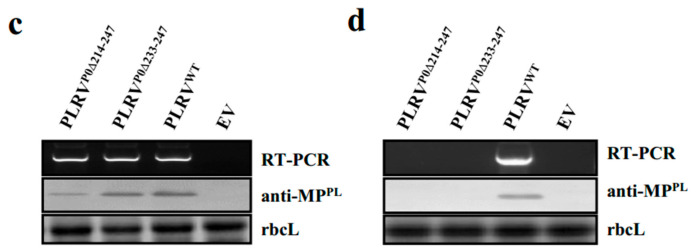
Infectivity analysis of C-terminal truncated mutants of P0^PL^ obtained from the full-length infectious cDNA clone of PLRV (pCB-PLRV) in *N. benthamiana*. (**a**) PLRV^P0∆214–247^ and PLRV^P0∆233–247^ mutation is incorporated into the P0 coding sequence without disrupting the P1 sequence. (**b**) Induction of cell death by C-terminal truncated mutants (PLRV^P0∆214–247^ and PLRV^P0∆233–247^) in agro-infiltrated *N. benthamiana* leaves at 7 dpi. Empty pCB vector (EV) and wild-type (PLRV^WT^) were used as negative and positive controls, respectively. (**c**) RNA and protein determinations from virus-inoculated *N. benthamiana* leaves at 3 dpi as assessed by RT-PCR using PLRV-specific primers and Western blotting analysis using a PLRV-MP-specific antibody (anti-MP^PL^). (**d**) RNA and protein determinations from the upper leaves of respective *N. benthamiana* plants at 14 dpi as assessed by RT-PCR using PLRV-specific primers and Western blotting analysis using a PLRV-MP-specific antibody (anti-MP^PL^). Stained rubisco is shown to indicate equal lane loading.

Based on the function screening results of PLRV P0 truncation mutants, two PLRV mutants cDNA clones, named PLRV^P0∆214–247^ and PLRV^P0∆233^^–247^ were successfully generated by introducing premature stop codon at position nt 710 or nt 767, respectively, without altering the translation products of the overlapped ORF1 (Figure 4a). The mutant PLRV^P0∆214–247^ encodes a truncated P0, which failed to suppress local or systemic RNA silencing, while the mutant PLRV^P0∆233^^–247^ encodes a truncated P0, which retained the local VSR activity but lost its systemic VSR activity.

To address the impact of the mutations on PLRV accumulation, *N. benthamiana* plants were agro-infiltrated with these mutants. At 7 dpi, PLRV mutant-infiltrated leaves showed cell death symptoms as did the positive control (wild-type PLRV), whereas the negative control (pCB empty vector) did not show any cell death. However, the wild-type exhibited more noticeable symptoms than the mutants (Figure 4b). Subsequent RT-PCR and immunoblot analyses showed that both mutants were detectable in inoculated leaves at 3 dpi. However, the mutant PLRV^P0∆214–247^ accumulated more at a lower level than the mutant PLRV^P0∆233–247^ and wild-type PLRV in the inoculated leaves (Figure 4c).

The RT-PCR results and Western blotting analyses using extracts from the upper leaves of the respective *N. benthamiana* plants confirmed that only the wild-type caused systemic infections at 14 dpi, and none of the mutants were able to infect upper leaves (Figure 4d).

To determine the impacts of these mutants on PLRV infectivity in its natural hosts, potato and black nightshade plants were inoculated with PLRV and its mutants. Interestingly, none of the mutants caused cell death in the inoculated leaves at 5 dpi, but the wild-type did. Similarly, RT-PCR and immunoblot analyses further confirmed that none of the mutants accumulated in inoculated leaves at 3 dpi or in infected upper leaves at 14 dpi (Figure S1), whereas wild-type PLRV could infect upper leaves of the inoculated natural hosts. These results demonstrated that a functional C-terminal region of P0 was required for long-distance movement of poleroviruses.

### 2.3. Neither Stable Nor Transient Ectopic Expressions of VSR Proteins Rescued Systemic Infections of BrYV or PLRV P0 C-Terminal Truncated Mutants

**Figure 5:**
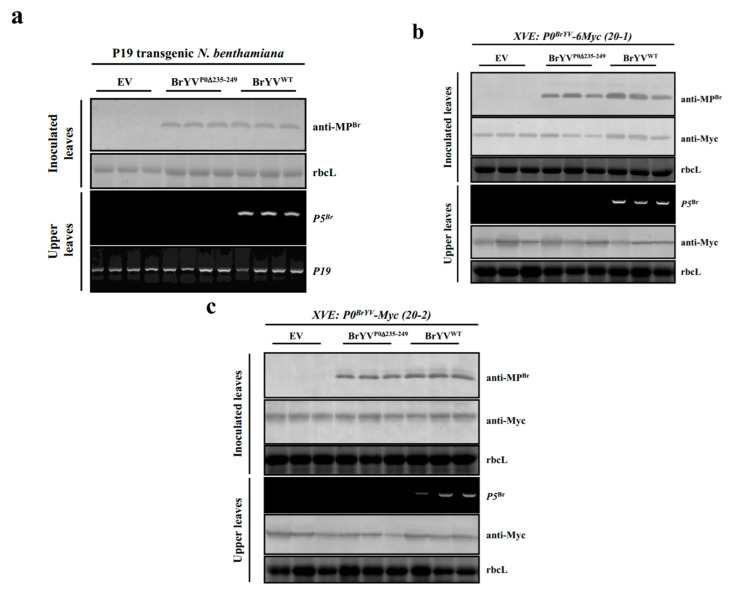
Stable P19 expression in transgenic plants or the heterologous stable expression of P0^Br^ protein did not rescue systemic infections by the BrYV C-terminal truncated mutant BrYV^P0∆235–249^. (**a**) Protein determinations from virus-inoculated P19 transgenic *N. benthamiana* leaves at 2 dpi as assessed by Western blotting analysis using a BrYV-MP-specific antibody (anti-MP^Br^). RNA determinations from the upper leaves of P19 transgenic *N. benthamiana* plants at 14 dpi as assessed by RT-PCR using P5^Br^- and P19-specific primers. (**b**,**c**) Protein determinations from virus-inoculated XVE: P0^Br^-6Myc transgenic *N. benthamiana* leaves at 2 dpi as assessed by Western blotting analysis using a Myc monoclonal antibody (anti-Myc) and a BrYV-MP-specific antibody (anti-MP^Br^). RNA determinations from the upper leaves of XVE: P0^Br^-6Myc transgenic *N. benthamiana* plants at 14 dpi as assessed by RT-PCR using P5^Br^-specific primers. Western blotting analysis of P0^Br^ in XVE: P0^Br^-6Myc transgenic *N. benthamiana* using a Myc monoclonal antibody (anti-Myc) at 14 dpi. Estradiol (100 μM) was sprayed on leaves to induce expression of P0^Br^-6Myc in transgenic *N. benthamiana* plants. 20-1 and 20-2 represent two transgenic lines.

To determine whether the systemic infection of mutant BrYV^P0∆235–249^ could be rescued by stable expression of a heterologous VSR protein, we tested the infection of BrYV^P0∆235–249^ in P19 (a VSR encoded by *Tomato bushy stunt virus*) transgenic *N. benthamiana* plants [34]. The accumulation level of BrYV^P0∆235–249^ in inoculated leaves was similar to that of wild-type BrYV as determined by Western blotting at 2 dpi. The wild-type BrYV systemically infected plants at 14 dpi, but the mutant BrYV^P0∆235–249^ was not able to infect upper leaves of inoculated plants (Figure 5a).

We then tested the infection of mutant BrYV^P0∆235–249^ in XVE: P0^Br^-6Myc transgenic *N. benthamiana* plants to determine whether the inducible stable expression of P0^Br^ could complement the systemic infection of BrYV^P0∆235–249^ [12]. At 2 dpi, the BrYV^P0∆235–249^ accumulation level in inoculated leaves was not significantly different from that of the wild-type virus as indicated by the Western blotting result. However, at 14 dpi, the mutant BrYV^P0∆235–249^ was not detected in upper leaves by RT-PCR with P5^Br^ gene-specific primers, although the wild-type BrYV could infect the upper leaves (Figure 5b,c).

In addition, the P0 C-terminal truncated PLRV mutants (PLRV^P0∆214–247^ and PLRV^P0∆233–247^) were co-infiltrated with a transient supplementary VSR, P0^PL^, into *N. benthamiana* plants. At 7 dpi, PLRV mutants co-infiltrated with P0^PL^ showed more prominent cell death symptoms than PLRV mutants alone (without P0^PL^). However, RT-PCR and Western blotting analyses showed that at 3 dpi, RNA and protein accumulation levels were comparable with those of wild-type in both mutants. Similar to the results of the BrYV mutant, PLRV^P0∆214–247^, after co-infiltration with a supplementary VSR (P0^PL^), failed to systemically infect *N. benthamiana* plants (Figure S2).

### 2.4. The Mutant BrYV^P0∆235–249^ Could Not Systemically Infect DCL2/4i or RDR6i Transgenic Plants

**Figure 6:**
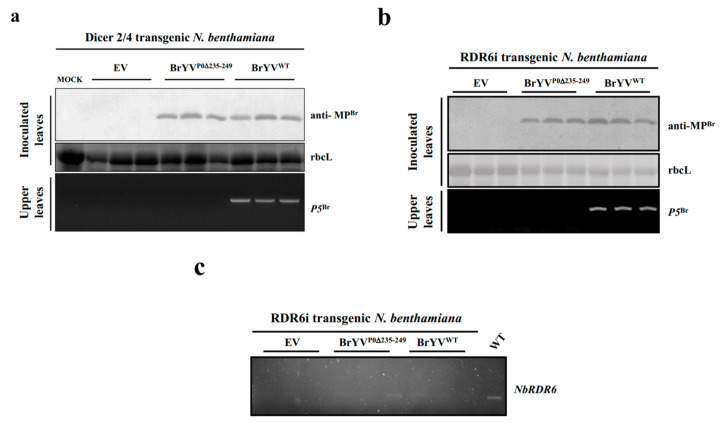
DCL2/4i and RDR6i transgenic plants were not systemically infected by the BrYV mutant BrYV^P0∆235−249^. (**a**,**b**) Protein determinations from virus-inoculated DCL2/4i and RDR6i transgenic *N. benthamiana* leaves at 2 dpi as assessed by Western blotting analysis using a BrYV-MP-specific antibody (anti-MP^Br^). RNA determinations from the upper leaves of RDR6i transgenic *N. benthamiana* plants at 14 dpi as assessed by RT-PCR using P5^Br^-specific primers. (**c**) Confirmation of RDR6i transgenic plants by RT-PCR using NbRDR6-specific primers. WT: non-transgenic *N. benthamiana* plants.

The Dicer-like proteins (DCLs) and RDR6 protein play important roles in the RNA-silencing pathway. In the RNA-silencing pathway, DCLs produce small RNA and induce the local and systemic RNAi response. Viral replication intermediates can indeed be processed in siRNA by Dicer proteins. The produced siRNAs are then loaded into AGO1 to form functional RISC, able to degrade new copies of viral RNA. In the downstream pathway, RDR6 acts as an amplification mechanism of siRNA production, by reverse transcribing aberrant viral RNAs into double-stranded RNAs, which are further used as a template for siRNA production [35]. DCL2 and DCL4 play essential roles in systemic PTGS in *N. benthamiana*. Inhibition of DCL2 and DCL4 expression reduced the spread of gene silencing [36]. To determine whether loss of DCL2/4 or RDR6 expression could compensate for systemic infections by the mutant BrYV^P0∆235–249^, we inoculated DCL2/4i [4] and RDR6i transgenic *N. benthamiana* [37] leaves with BrYV^P0∆235–249^. At 2 dpi, the accumulation of BrYV^P0∆235–249^ in inoculated leaves was similar to that of the wild-type BrYV as assessed by immunoblot detection of MP^Br^. However, at 14 dpi, the mutant BrYV^P0∆235–249^ was not detected in upper leaves, although the wild-type BrYV could infect the upper leaves (Figure 6). These results demonstrated that DCL2/4i and RDR6i transgenic plants did not rescue systemic infections of the mutant BrYV^P0∆235−249^.

### 2.5. The P0^Br^ Defective Mutant P0^Br∆235–249^ Showed No Significant Differences in AGO1-Degradation Induction, Subcellular Localization or NbSKP1 and AtRAF2 Interactions, Compared with P0^Br^ Wild-Type

**Figure 7:**
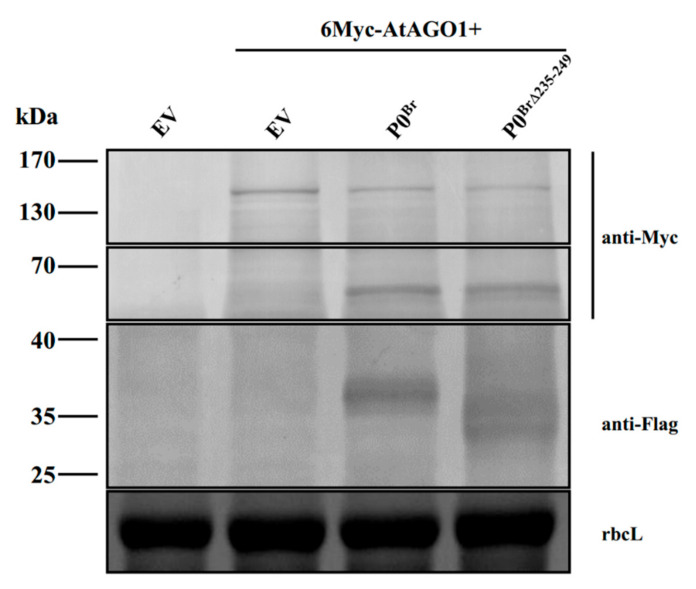
The P0^Br^ defective mutant P0^Br∆235–249^ had the ability to induce AGO1-degradation induction, the same as P0^Br^. Analysis of the AGO1-degradation capability of P0^Br∆235–249^. The 6Myc-tagged AGO1 was co-infiltrated into leaf patches of *N. benthamiana* with P0^Br∆235–249^, P0^Br^ or EV. Western blotting analyses of 6Myc-tagged AGO1 and 3Flag-tagged P0^Br^ or P0^Br∆235–249^ using a Myc monoclonal antibody (anti-myc) and a Flag monoclonal antibody (anti-Flag), respectively. Stained rubisco is shown to indicate equal lane loading.

Previously, we revealed that P0^Br^ had an impact on AGO1 destabilization. In the presence of P0^Br^, the accumulation of AGO1 decreases [12]. To determine if P0^Br∆235–249^ was impaired in its activity for mediating AGO1 destabilization, AGO1 and P0^Br∆235–249^ were co-expressed in *N. benthamiana* through agro-infiltration and protein samples were analyzed at 2 dpi. The results showed that a decrease in AGO1 levels associated with P0^Br∆235–249^ was similar to the wild-type P0^Br^ (Figure 7).

Previous studies showed that P0^Br^ localizes to the nucleus and cytoplasm [28]. Therefore, we were interested in finding out whether there was a difference in the subcellular localization of P0^Br∆235–249^ and P0^Br^. To observe the subcellular localization of P0^Br∆235–249^, the mutant was infiltrated into *N. benthamiana*. At 2 dpi, confocal microscopy showed that the subcellular localization of P0^Br∆235–249^ was similar to that of P0^Br^ in *N. benthamiana*, localizing to the nucleus and the cytoplasm (Figure S3).

In addition, we previously found that P0^Br^ interacted with NbSKP1 and AtRAF2 [12,28]. Similarly, we want to know whether there are differences in NbSKP1 and AtRAF2 interactions. Here, we examined interactions by a yeast two-hybrid system. P0^Br^ and P0^Br∆235–249^ were cloned into bait vector pGBKT7 and transformed into yeast Y187. NbSKP1 and AtRAF2 were cloned into prey vector pGADT7 and transformed into yeast AH109. The interaction between P0^Br^ and NbSKP1 served as the positive control [12]. The results showed that P0^Br∆235–249^ also interacted with these proteins as the wild-type P0^Br^ (Figure S3).

### 2.6. The Systemic Infection Capability of the Mutant BrYV^P0∆235–249^ Was Rescued Partially by Co-Infection with PLRV

**Figure 8:**
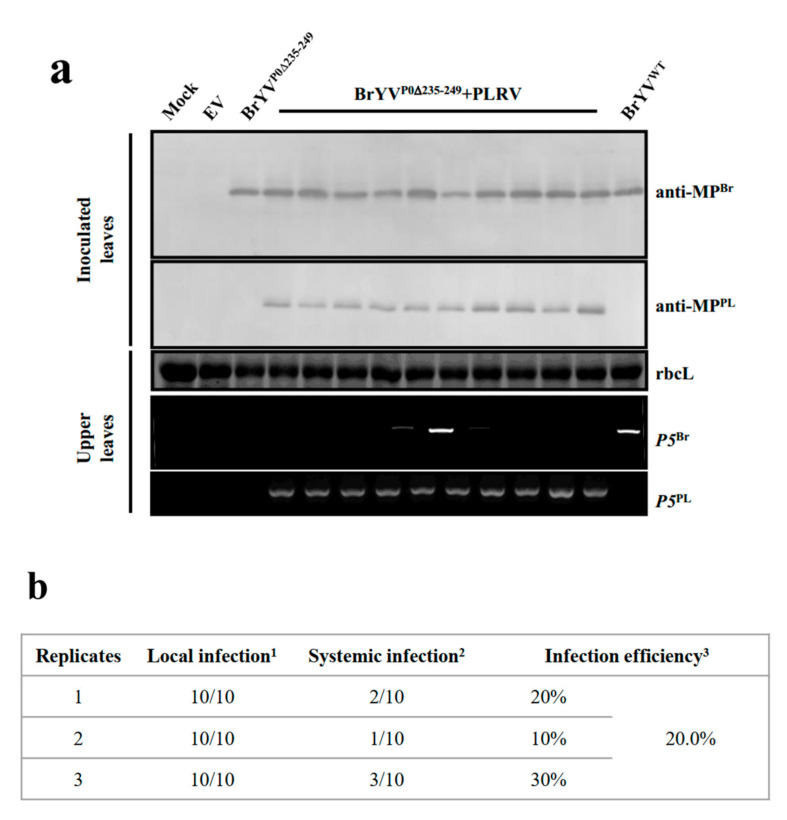
The capability of BrYV mutant BrYV^P0∆235–249^ to produce a systemic infection was rescued partially by co-infection with PLRV. (**a**) Protein determinations from virus-inoculated *N. benthamiana* leaves co-expressed with PLRV at 2 dpi as assessed by Western blotting analysis using a BrYV-MP-specific antibody (anti-MP^Br^) and a PLRV-MP-specific antibody (anti-MP^PL^). RNA determinations from the upper leaves of *N. benthamiana* plants at 14 dpi as assessed by RT-PCR. (**b**) Summary of the infection efficiency of BrYV^P0∆235–249^ with the help of PLRV. Determination in (**a**) were repeated three times to calculate the BrYV^P0∆235–249^ infection efficiency of the inoculated leaves and the systemically infected leaves. ^1^ The proportion of BrYV^P0∆235–249^ detected in the inoculated leaves at 2 dpi. ^2^ The proportion of BrYV^P0∆235–249^ detected in the upper leaves at 14 dpi. ^3^ The proportion of successful systemic infections by BrYV^P0∆235–249^.

Since BrYV^P0∆235–249^ cannot infect upper leaves, we tested whether BrYV^P0∆235–249^ could systemically infect *N. benthamiana* with the help of PLRV, which also belongs to polerovirus. We co-inoculated *N. benthamiana* with BrYV^P0∆235–249^ and PLRV by agro-infiltration. At 2 dpi, BrYV^P0∆235–249^ and PLRV could be detected by Western blotting in inoculated leaves. At 10 dpi, an RT-PCR analysis result showed that PLRV was detected in the upper leaves of all the 10 plants, and BrYV^P0∆235–249^ was detected in three plants among them (Figure 8), indicating that PLRV could partially rescue the systemic infection of mutant BrYV^P0∆235–249^ in *N. benthamiana*.

### 2.7. The Mutant P0^Br∆235–249^ but Not the P0^PL∆233–247^ Had a Relatively Weaker Interaction with MP Than the Wild-Type P0

**Figure 9:**
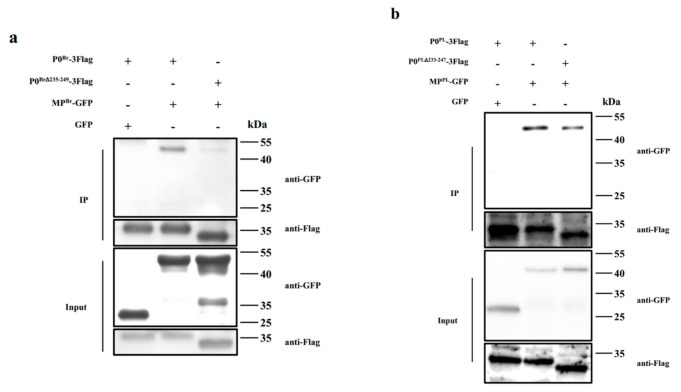
Both the P0 C-terminal truncated mutants and wild types of BrYV and PLRV interacted with their MPs. (**a**) Co-immunoprecipitation analyses of P0^Br^ and MP^Br^ proteins in *N. benthamiana* leaves. P0^Br^-3Flag was co-expressed with MP^Br^ in *N. benthamiana*. P0^Br^-3Flag was co-expressed with MP^Br^ in *N. benthamiana*. P0^Br^-3Flag and GFP were used as negative controls. (**b**) Co-immunoprecipitation analyses of P0^PL^ and MP^PL^ proteins in *N. benthamiana* leaves. P0^PL^-3Flag was co-expressed with MP^PL^ in *N. benthamiana*. P0^PL^-3Flag and GFP were used as negative controls. Protein complexes were immunoprecipitated using anti-Flag beads. The co-immunoprecipitated proteins were probed with a GFP antibody (anti-GFP) and a Flag monoclonal antibody (anti-Flag).

According to the above results, we were able to show unambiguously that P0 participates in the systemic movement of BrYV. We wondered whether P0^Br^ was related to other movement-related proteins encoded by BrYV, so a co-immunoprecipitation assay was performed. P0^Br^-3Flag was transiently co-expressed with MP^Br^-GFP in *N. benthamiana* and protein extracts were immunoprecipitated by anti-Flag beads. The result revealed that P0^Br^ interacted with MP^Br^ (Figure 9a).

To further test whether P0^Br∆235–249^ could interact with MP^Br^, we also performed the co-immunoprecipitation assay with MP^Br^-GFP and the mutant P0^Br∆235–249^-3Flag. Interestingly, the co-IP analysis result showed that P0^Br∆235–249^ had a relatively weaker interaction with MP^Br^ compared with wild-type P0^Br^, as shown in Figure 9a. However, P0^PL^ and its mutant P0^PL∆233–247^ showed no significant difference in the interaction with MP^PL^ (Figure 9b), implying that this interaction may not be the key cause of preventing the virus from systemically infecting.

## 3. Discussion

The P0 proteins of poleroviruses play important roles in the suppression of the plant’s RNA silencing activity by inducing an ER-derived autophagy degradation of membrane-bound AGO1 [21,22]. In our previous studies, we revealed that P0 of BrYV is an RNA silencing suppressor and is indispensable for efficient systemic infection of BrYV [12]. Chiba et al. also reported that RNA-silencing suppressor of *Beet necrotic yellow vein virus* was essential for long-distance movement [24]. In this study, we constructed various C-terminal truncation mutants of BrYV and PLRV P0 and investigated their functions. Our result indicated that 15 amino acids in the C-terminus of P0^Br^ (P0^Br∆235–249^) and P0^PL^ (P0^PL∆233**−**247^) are essential for the systemic VSR activity but not for local VSR activity (Figure 1 and Figure 2). Interestingly, an infectivity analysis in *N. benthamiana* plants revealed that both BrYV and PLRV mutants (BrYV^P0∆235–249^ and PLRV^P0∆233–247^) were detectable in the inoculated leaves but not in the upper leaves of inoculated plants (Figure 3 and Figure 4). Surprisingly, this study also showed that neither mutants of PLRV (PLRV^P0∆214–247^ and PLRV^P0∆233–247^) accumulated in the inoculated leaves nor the systemic leaves of potato or black nightshade plants, which are the natural hosts of PLRV, at 3 and 14 dpi, respectively. Only wild-type PLRV was detected in the infiltrated leaves of its natural hosts at a lower concentration than in *N. benthamiana* leaves, at 3 dpi (Figure S1), consistent with the findings of Alvarez and Derrick [38,39]. The inability of PLRV mutants to infect their natural hosts may be due to host immune responses, which is consistent with the report of Takahashi [40]. Thus, these results clearly illustrated the important role of C-terminal regions of P0 in BrYV and PLRV in the long-distance movement of viruses. It was also hypothesized that the systemic VSR activity levels of P0s might be associated with systemic viral infections.

To verify this hypothesis, we used transgenic *N. benthamiana* plants expressing P19 or P0 to complement the systemic VSR activity of the mutant BrYV^P0∆235–249^. P19 transgenic *N. benthamiana* plants stably express the P19 protein, and P19 itself, as a classic VSR has a systemic suppressor function [34]. XVE: P0^Br^-6Myc transgenic *N. benthamiana* plants can also supply the systemic VSR activity through the inducible expression of the P0 protein [12]. However, even with the help of ectopic VSRs, the mutant BrYV^P0∆235–249^ failed to infect the upper leaves of inoculated plants. Consistently, co-infiltration of the mutant PLRV^P0∆233–247^ with an additional VSR (P0^PL^) did not regain the systemic infection (Figure S2). Rashid et al. also observed that the co-infiltration of PLRV-P0 VSR defective mutants in the three essential motifs (F-box-like motif, G139/W140/G141-like motif and the C-terminal conserved region) with an additional VSR (P0^PL^) did not produce a successful systemic infection [27]. These results indicated that the lack of systemic VSR activity from the C-terminal region-deleted P0 may not be the key cause of the loss of systemic viral infection.

RNA silencing in plants can protect the host from virus infection. In the antiviral RNA-silencing pathway, DCLs play important roles as conserved dsRNA endoribonucleases in RNAi and post-transcriptional gene silencing [35]. DCL2 can promote cell-to-cell spread of PTGS, and DCL4 inhibits the cell-to-cell spread of virus-induced gene silencing. Inhibition of DCL2 and DCL4 expression has been shown to reduce systemic PTGS [36]. RDRs are required to amplify double-stranded RNA from template single-stranded RNA in the RNA-silencing pathway, and the RNAi pathway can directly target viruses for degradation without the help of RDR6 [41]. DCLs and RDRs play key roles in the plant’s antiviral RNA-silencing pathway [7,37]. To determine the systemic infection of the BrYV^P0∆235–249^ mutant in silencing-deficient plants, we inoculated DCL2/4i or RDR6i transgenic *N. benthamiana* with the mutant BrYV^P0∆235–249^. Our results indicated that even if the DCLs and RDR6 antiviral pathway were inhibited, the mutant BrYV^P0∆235–249^ was still unable to systemically infect *N. benthamiana* plants (Figure 6). In RDR6i transgenic *N. benthamiana* plants, it was possible that there was still sufficient RDR6 expression in the knock-down transgenic plants, which is enough for blocking viral systemic infection. Thus, it was likely that P0 involved in the systemic spread of the virus is related to other proteins in the RNAi pathway.

AGO1 also plays an antiviral role in plants. Recent studies show that P0 can promote degradation of AGO1 by autophagy and ubiquitylation. P0 from polerovirus as an F-box protein was reported to interact with SKP1 [20,21,22]. Our previous results found that P0 of BrYV interacts with SKP1 to facilitate self-stability and mediates the decay of AGO1 [12]. In this work, the P0^Br^ mutant P0^Br∆235–249^ still induced degradation of AGO1 (Figure 7) and interacted with SKP1 (Figure S3), which shows no difference from the wild type P0^Br^. As we reported previously, P0^Br^ interacts with host factor RAF2 to impair its antiviral activity [28]. However, this work shows that the P0^Br^ mutant P0^Br∆235–249^ also interacts with RAF2 (Figure S3). The P0 roles in stability of viral and host components involved in systemic viral infection require further investigation.

Additionally, a synergistic effect may occur between viruses during viral mixed infections. In synergistic interactions, one virus may complement the defect in the intercellular or long-distance movement of another virus [42]. For instance, P3N-PIPO, the MP of *Clover yellow vein virus*, enhances *White clover mosaic virus* virulence by facilitating its spread [43]. Here, when the mutant BrYV^P0∆235–249^ was co-infected with PLRV, 20.0% of plants were detected BrYV^P0∆235–249^ (Figure 8). BrYV and PLRV belong to the same family, and PLRV can complement BrYV^P0∆235–249^ in reaching the vasculature, where PLRV can further assist the replication of BrYV^P0∆235–249^, leading to a systemic spread. However, the detailed synergistic mechanism should be further investigated.

At the site of initial infection, a virus mainly exists in the epidermal and mesophyll cells, where it is replicated and translated. Then, the virus moves to neighboring cells via plasmodesmata for more replication and translation [44]. Viruses then pass-through bundle sheathes, vascular parenchyma and companion cells to enter sieve elements. Finally, the virus is transported to other plant tissues through sieve elements to infect the entire plant [45]. The MPs of viruses are essential for their cell-to-cell and long-distance movement. For instance, the mutant of BYDV-PAV lacking the capacity to produce MP was unable to infect plants, indicating that MP of BYDV-PAV provides a systemic movement function [46]. Two PLRV mutants were cloned to investigate P17 movement protein functions in a long-distance movement. Although P17 mutants replicated and accumulated in inoculated leaves of potato and *P. floridana*, they were unable to complete a systemic infection [33]. We speculated that the P0 protein functioned together with other viral proteins or host factors to produce systemic movement, and the C-terminal peptides of P0 are required for these interactions. After some exploration, we found that MP^Br^ interacted with P0^Br^, and MP^Br^ and P0^Br∆235–249^ had a weaker interaction compared with P0^Br^; however, P0^PL^ and its mutant P0^PL∆233–247^ had no significant difference in the interaction with MP^PL^ (Figure 9). Based on the above results, we did not propose a reasonable speculation that C-terminal amino acid residues of P0 affected the interaction between MP and P0 of poleroviruses, which in turn caused mutant virus to be unable to spread systematically. Thus, searching and analyzing the complex interaction between P0 and other proteins may provide more explanation about the mechanism behind this phenomenon.

## 4. Materials and Methods

### 4.1. Plant Materials and Growth Conditions

Wild-type *Nicotiana benthamiana* and a transgenic green fluorescent protein (GFP)-containing *N. benthamiana* 16c line were grown in a greenhouse with a 16-h light/8-h dark photoperiod at 25 ± 1 °C and 60% relative humidity [47]. The same growth conditions were provided for P0^Br^-6Myc-, P19-, DCL2/4i-, RDR6i-transgenic *N. benthamiana*, potato (variety’ Lalpakri’) and black nightshade plants.

### 4.2. Plasmid Construction

The binary vectors pGD, pGDG and pGDP19 [14,28] were used for transient expression experiments. Construction of pGD-P0^Br^-3Flag and pGD-P0^PL^-3Flag were described by Sun and Rashid [27,28]. For P0’s subcellular localization, P0^Br^ and P0^Br∆235–249^ were cloned into pSuper 1300-GFP [48]. C-terminal truncation mutants of P0^Br^ and P0^PL^ were produced by inverse PCR with specific primer pairs having *Xho*I and *Apa*I sites, respectively, as described previously by Rashid [27].

P0 C-terminal truncation mutants of full-length pCB-BrYV (BrYV) were generated using pTBrYV by inverse PCR and subsequent ligation with the predigested pCB301 vector.

A full-length PLRV infectious cDNA clone, pCB-PLRV, was generated in the pCB301 binary vector from pBNUP110, a Canadian isolate of PLRV [49] by inverse PCR. pCB-PLRV (PLRV) was further amplified using specific primers containing *Apa*I and *Spe*I sites, respectively, and subsequently inserted into the pMD19-T (simple) vector (TaKaRa, Shiga, Japan) to generate pTPLRV. P0 C-terminal truncation mutants of full-length pCB-PLRV were generated from pTPLRV by inverse PCR and subsequent ligation with the predigested pCB301 vector. All the constructs were validated by PCR amplification and sequencing. All of primers are listed in Table S1.

### 4.3. Plant Agro-Infiltration

*Agrobacterium tumefaciens* strain C58C1 was transformed independently with each plasmid construct using the freeze–thaw method as described by Holsters [50]. Co-infiltration assays were performed as described previously by Zhuo [14], and an infectivity analysis was performed as described by Ruiz [51]. *Agrobacterium* suspensions were adjusted to OD600 = 0.5 for each culture.

The GFP fluorescence in leaves was illuminated using a BLAK-RAY long-wave UV lamp (B 100AP/R, Upland, CA, USA), and photographs were taken using a Canon digital camera (EOS 550D, Tokyo, Japan) using a yellow filter (Kodak Wratten gelatin filter, no. 15). Both 3-day post-infiltration (dpi) local leaves and 14-dpi systemically infected leaves were harvested for viral RNA and protein assays. Each experiment was repeated at least three times.

### 4.4. RNA Extraction, Reverse Transcriptase PCR and Northern Blotting Analyses

Total RNAs harvested using the sodium dodecyl sulfate (SDS)-phenol-chloroform method [52] from the locally and systemically infected leaves of the infiltrated plants were templates for the synthesis of first-strand cDNAs with specific primers and Moloney murine leukemia virus (M-MLV) reverse transcriptase (Promega, Fitchburg, WI, USA). Reverse transcriptase PCR (RT-PCR) was performed as described previously by Zhang [32] using specific primers with synthesized cDNAs serving as templates. Northern blotting analysis was performed as described previously by Zhang [32].

### 4.5. Protein Extraction and Western Blotting Analyses

Total proteins were harvested from leaf samples as described previously by Sun et al. (2018) using 2× SDS loading buffer (100 mm Tris (pH 6.8), 4% (*w*/*v*) SDS, 20% (*v*/*v*) glycerol and 0.2% (*w*/*v*) bromophenol blue) containing 10% β-mercaptoethanol. Proteins were isolated on 12.5% SDS polyacrylamide gels and then transferred onto polyvinylidene fluoride membranes (Ge Healthcare, Buckinghamshire, UK). The membranes were blotted with a rabbit anti-flag antibody (1:1000; Sigma–Aldrich, St. Louis, MO, USA), mouse anti-flag antibody (1:5000; Sigma–Aldrich), rabbit anti-GFP antibody (1:3000; Genscript, Nanjing, China) or rabbit anti-PLRV MP antibody (1:5000) [53]. Immunoreactive proteins were successively visualized by blotting with goat anti-rabbit AP antibody (1:10,000; Sigma–Aldrich), goat anti-mouse AP antibody (1:10,000; Sigma–Aldrich), goat anti-rabbit HRP antibody (1:3000; Sigma–Aldrich) or goat anti-mouse HRP antibody (1:3000; Bio-Rad, Hercules, CA, USA) followed by visualization using nitro-blue tetrazolium and 5-bromo-4-chloro-3-indolyphosphate (Sigma–Aldrich) or a chemiluminescence detection kit (GE Healthcare).

### 4.6. In Vivo Co-Immunoprecipitation

Co-immunoprecipitation was performed as previously described [12].

### 4.7. GAL4 Yeast Two-Hybrid Assay

The matchmaker GAL4 two-hybrid system 3 was used in yeast two-hybrid (Y2H). Protein interactions were tested by yeast mating assay on synthetic dropout (SD) media lacking Trp and Leu and SD media lacking Ade, His, Trp and Leu at 30 °C for 3–5 days.

## Data Availability

Not applicable.

## Supplementary Materials

The following are available online at https://www.mdpi.com/article/10.3390/ijms23041945/s1.
