# Supplementary material for: The Carboxyl Terminal Regions of P0 Protein Are Required for Systemic Infections of Poleroviruses
Source: Int J Mol Sci. 2022 Feb 9;23(4):1945. doi: 10.3390/ijms23041945 (PMC8875975; doi:10.3390/ijms23041945)
Supplement: Supplementary file 1 [file ijms-23-01945-s001.zip › ijms-1497627-supplementary.pdf]

Supporting Information

Figure S1

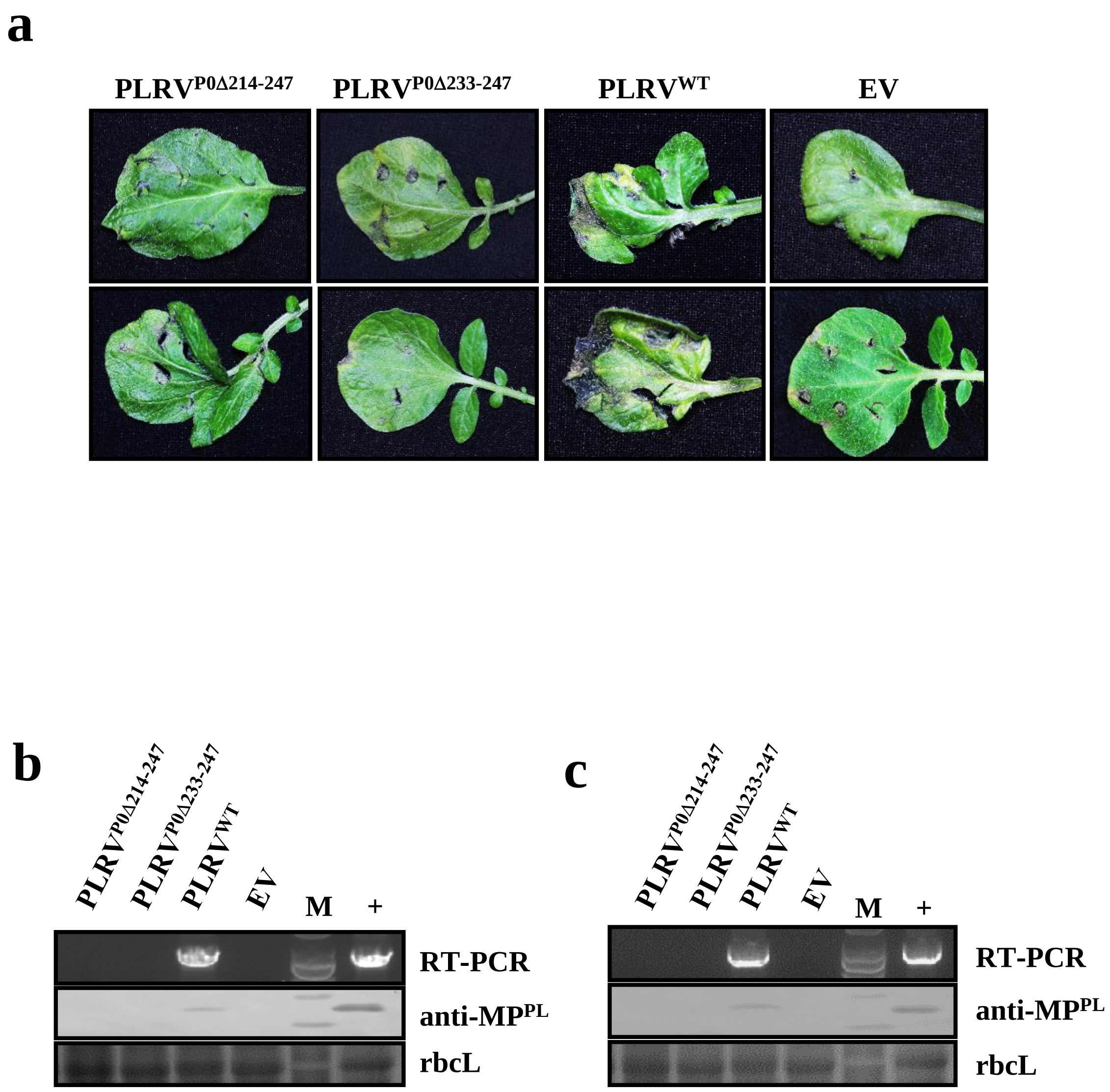

d

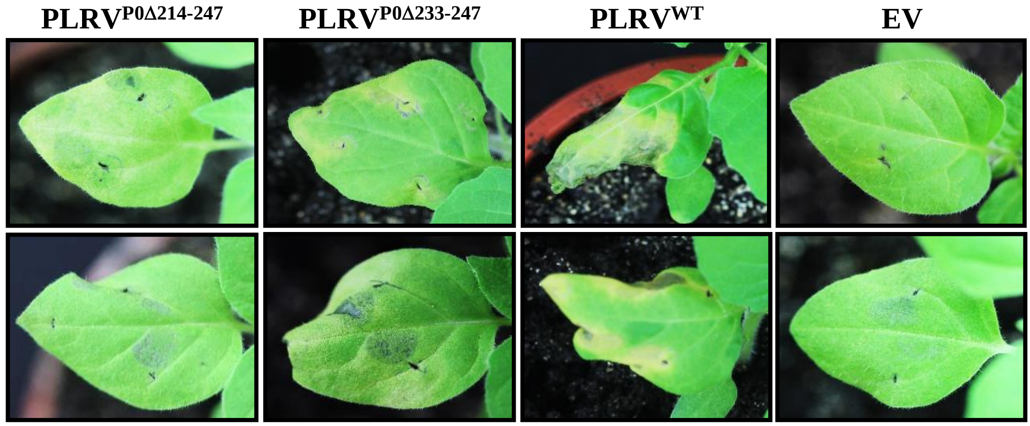
PLRV$^{P0\Delta 214-247}$
PLRV$^{P0\Delta 233-247}$
PLRV^{WT}
EV

e

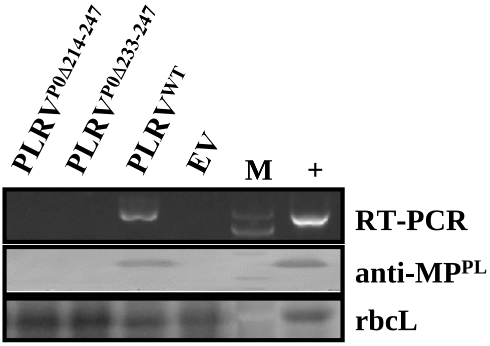
PLRV$^{P0\Delta 214-247}$
PLRV$^{P0\Delta 233-247}$
PLRV^{WT}
EV
M
+
RT-PCR
anti-MP^{PL}
rbcL

f

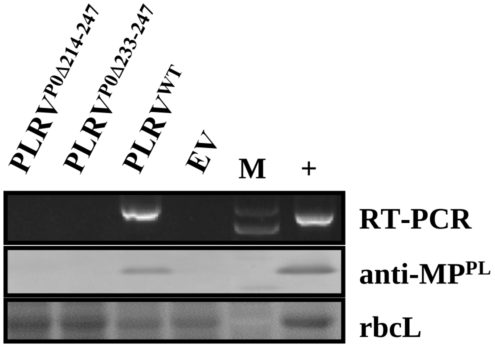
PLRV$^{P0\Delta 214-247}$
PLRV$^{P0\Delta 233-247}$
PLRV^{WT}
EV
M
+
RT-PCR
anti-MP^{PL}
rbcL

Figure S1. Infectivity analysis of C-terminal truncated mutants of P0^{PL} obtained from the full-length infectious cDNA clone of PLRV (pCB-PLRV) in potato and black nightshade plants.

(a) Induction of cell death by C-terminal truncated mutants (PLRV$^{P0\Delta214–247}$ and PLRV$^{P0\Delta233–247}$) in agro-infiltrated potato (variety 'Lalpakri') leaves at 5 dpi. Empty pCB vector (EV) and wild-type (PLRV^{WT}) were used as negative and positive controls, respectively.

(b) RNA and protein determinations from virus-inoculated potato leaves at 3 dpi as assessed by RT-PCR using PLRV-specific primers and a western blotting analysis using a PLRV-MP specific antibody (anti-MP^{PL}).

(c) RNA and protein determinations from the upper leaves of respective potato plants at 14 dpi as assessed by RT-PCR using PLRV-specific primers and a western blotting analysis using a PLRV-MP specific antibody (anti-MP^{PL}). PLRV infected *N. benthamiana* leaves were used as a positive control. Stained rubisco is shown to indicate equal lane loading.

(d) Induction of cell death by C-terminal truncated mutants ($PLRV^{P0\Delta214–247}$ and $PLRV^{P0\Delta233–247}$) in agro-infiltrated black nightshade leaves at 5 dpi. Empty pCB vector (EV) and wild-type ($PLRV^{WT}$) were used as negative and positive controls, respectively.

(e) RNA and protein determinations from virus-inoculated black nightshade leaves at 3 dpi as assessed by RT-PCR using PLRV-specific primers and a western blotting analysis using a PLRV-MP-specific antibody (anti-MP^{PL}).

(f) RNA and protein determinations from the upper leaves of respective black nightshade plants at 14 dpi as assessed by RT-PCR using PLRV-specific primers and a western blotting analysis using a PLRV-MP-specific antibody (anti-MP^{PL}). PLRV infected *N. benthamiana* leaves were employed as a positive control. Stained rubisco is shown to indicate equal lane loading. M: a DNA marker.

Figure S2

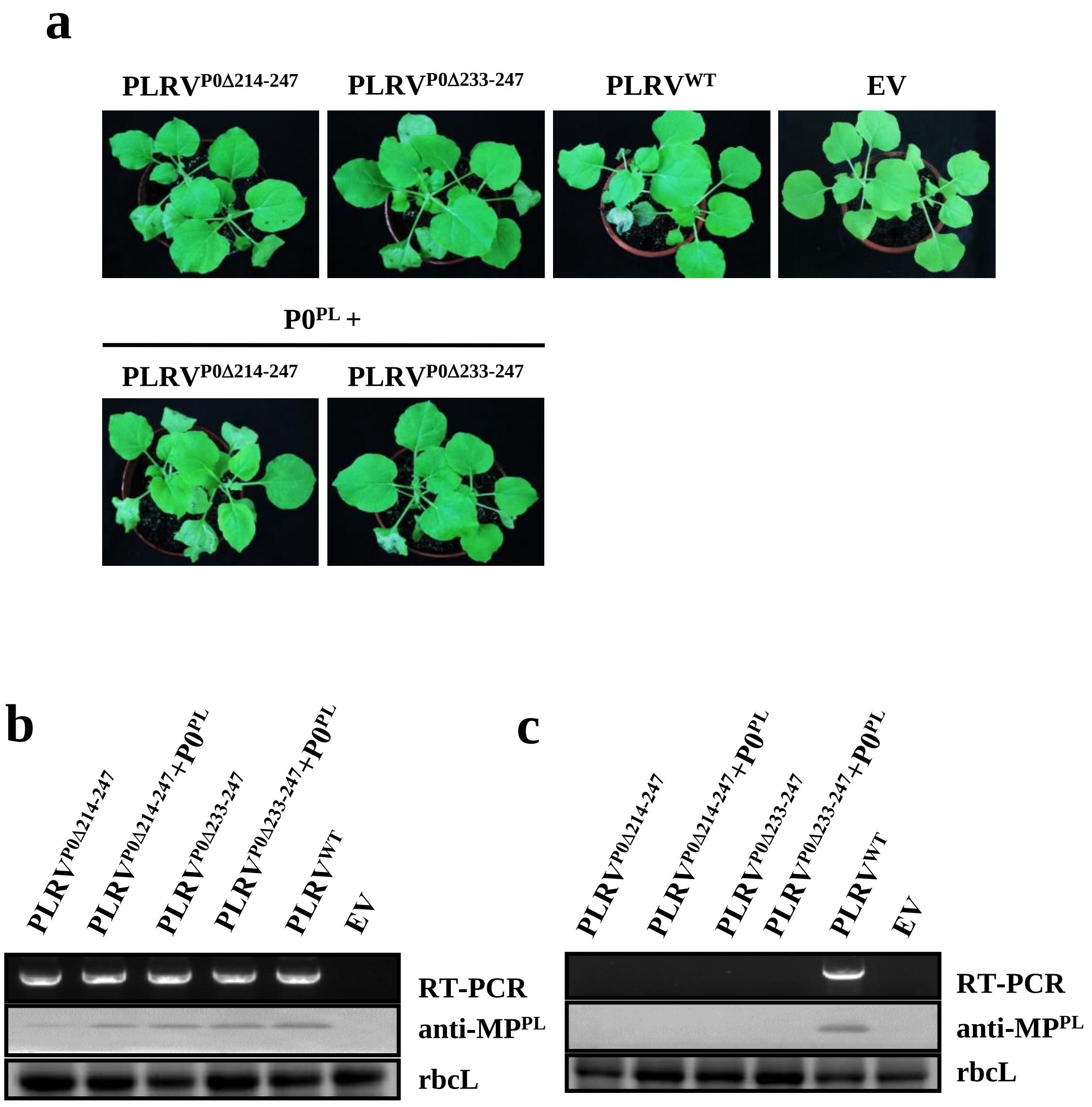
a
PLRV P0Δ214-247
PLRV P0Δ233-247
PLRV WT
EV
P0PL +
PLRV P0Δ214-247
PLRV P0Δ233-247
b
PLRV P0Δ214-247
PLRV P0Δ214-247+P0PL
PLRV P0Δ233-247
PLRV P0Δ233-247+P0PL
PLRV WT
EV
RT-PCR
anti-MPPL
rbcL
c
PLRV P0Δ214-247
PLRV P0Δ214-247+P0PL
PLRV P0Δ233-247
PLRV P0Δ233-247+P0PL
PLRV WT
EV
RT-PCR
anti-MPPL
rbcL

Figure S2. Effects of co-expression of a supplementary VSR (P0^{PL}) on infection by C-terminal truncated mutants of the P0^{PL} protein obtained from pCB-PLRV in *N. benthamiana*.

(a) Induction of cell death by C-terminal truncated mutants (PLRV$^{P0\Delta214–247}$ and PLRV$^{P0\Delta233–247}$) in agro-infiltrated *N. benthamiana* leaves alone or co-infiltrated with P0^{PL} at 7 dpi. Empty pCB vector (EV) and wild-type (PLRV^{WT}) were used as negative and positive controls, respectively.

(b) RNA and protein determinations from virus-inoculated *N. benthamiana* leaves at 3 dpi as assessed by RT-PCR using PLRV-specific primers and a western blotting analysis using a PLRV-MP-specific antibody (anti-MP^{PL}).

(c) RNA and protein determinations from the upper leaves of respective *N. benthamiana* plants at 14 dpi as assessed by RT-PCR using PLRV-specific primers and a western blotting analysis using a PLRV-MP-specific antibody (anti-MP^{PL}). Stained rubisco is shown to indicate equal lane loading.

Figure S3

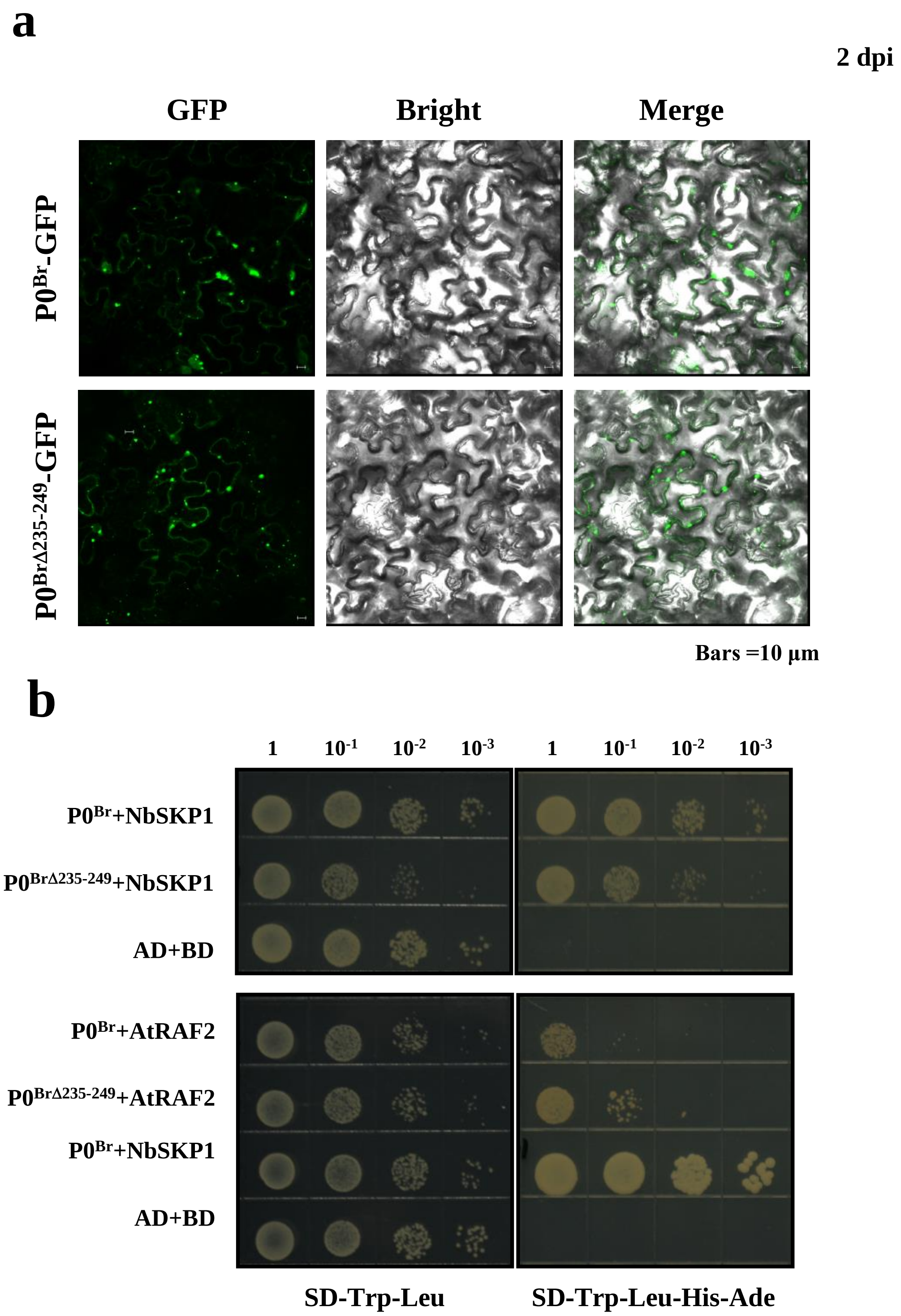
a
2 dpi
GFP
Bright
Merge
P0Br-GFP
P0BrΔ235-249-GFP
Bars =10 μm
b
1
10-1
10-2
10-3
P0Br+NbSKP1
P0BrΔ235-249+NbSKP1
AD+BD
P0Br+AtRAF2
P0BrΔ235-249+AtRAF2
P0Br+NbSKP1
AD+BD
SD-Trp-Leu
SD-Trp-Leu-His-Ade

Figure S3. The P0^{Br} defective mutant P0$^{Br\Delta 235–249}$ was not significantly different in subcellular localization or NbSKP1 and AtRAF2 interactions, compared with wild-type P0^{Br}.

(a) The subcellular localization of P0$^{Br\Delta 235–249}$ was similar to that of P0^{Br} in *N. benthamiana*. P0$^{Br\Delta 235-249}$ localized to the nucleus and the cytoplasm, as did P0^{Br}. Bars = 10 μm.

(b) Analysis of interactions between P0$^{Br\Delta 235–249}$ and NbSKP1 or AtRAF2 in the yeast two-hybrid system. The interactions of P0^{Br}/NbSKP1 and P0^{Br}/AtRAF2 served as positive controls. The interactions of pGBKT7(BD) and pGADT7(AD) served as negative controls.

Table S1. List of primers used in this study.

Primer	Sequence (5′ to 3′)	Application
**BrYV1-234F**	TAAGAAATCCTTTTCAGCTCTTCGGTC	Cloning for mutant
**BrYV1-234R**	AGGAGTGAGAAAGCAATCGAAATGATCC	Cloning for mutant
**BrYVP5-F**	ATGA ACTGGACCAA CGTGGACGCC CGAT	RT-PCR
**BrYVP5-R**	CTATTTCCTG AAAGAGAGGA AACCTTTC	RT-PCR
**BrYVP5-mR**	GTCCTCAGAA CCAGCATCTG AGAAACCT	RT-PCR
**PLP0-XF**	TATCTCGAGATGATTGTAT TGACCCAG	Cloning for mutant
**PLP0TR210R**	GGGCCCCTGGTTATACAACCGAGCAA	Cloning for mutant
**PLP0TR218R**	GGGCCCCTTAGCGCGCCCTTGTAGAT	Cloning for mutant
**PLP0TR237R**	GGGCCCCTCCAAATAATCTTCAGAGG	Cloning for mutant
**PLP0TR222R**	GGGCCCTGTTCTGAAAGACTTAGCGC	Cloning for mutant
**PLP0TR225R**	GGGCCCACCGGTAAGTGTTCTGAAAG	Cloning for mutant
**PLP0TR228R**	GGGCCCAATAGGAAAACCGGTAAGTG	Cloning for mutant
**PLP0TR219R**	GGGCCCAGACTTAGCGCGCCCTTGTA	Cloning for mutant
**PLP0TR220R**	GGGCCCGAAAGACTTAGCGCGCCCTT	Cloning for mutant
**PLP0TR221R**	GGGCCCTCTGAAAGACTTAGCGCGCC	Cloning for mutant
**PLTR214-apai-R**	GGGCCCTAGATCAAGCTGGTTATACA	Cloning for mutant
**PLTR233-apai-R**	GGGCCCAGAGGGGACATAAATAGGAA	Cloning for mutant
**P0PLΔ214-F**	TAAGGGCGCGCTAAGTCTTT	Cloning for mutant
**P0PLΔ214-R**	TAGATCAAGCTGGTTATACA	Cloning for mutant
**P0PLΔ233-F**	TAAGATTATTTGGAGGGCAG	Cloning for mutant
**P0PLΔ233-R**	AGAGGGGACATAAATAGGAA	Cloning for mutant
**PLRV5-28F**	ACAAAAGAATACCAGGAGAAATTGCAGC	Cloning for PLRV
**PLRVKp3R**	AAGGTACCACTACACAACCCTGTAA	Cloning for PLRV
**PLRV2723F**	CTTCAAAAGGTGTCAGGAG	Cloning for PLRV
**PLRV3656R**	GCCTGCGAAGGGATTG	Cloning for PLRV
**PLRV/Apa I-F**	GGGCCCTACCATCGTCATTA	Cloning for PLRV
**PLRV/Spe I-R**	ACTAGTATGGAGATATCATT	Cloning for PLRV
